# Sacral nerve stimulation in patients with ileal pouch-anal anastomosis

**DOI:** 10.1007/s00384-021-03981-z

**Published:** 2021-06-23

**Authors:** C. Seifarth, N. Slavova, C. Degro, K. S. Lehmann, M. E. Kreis, B. Weixler

**Affiliations:** Department of General, Visceral, and Vascular Surgery, Berlin Institute of Health, Charité Universitätsmedizin Berlin, Freie Universität Berlin, Humboldt-Universität Zu Berlin, Berlin, Germany

**Keywords:** Fecal incontinence, Ulcerative colitis, Sacral nerve stimulation, IPAA, Proctocolectomy

## Abstract

**Purpose:**

Functional results after proctocolectomy and ileal pouch-anal anastomosis (IPAA) are generally good. However, some patients suffer from high stool frequency or fecal incontinence. Sacral nerve stimulation (SNS) may represent a therapeutic alternative in these patients, but little is known about indication and results. The aim of this study was to evaluate incontinence after IPAA and demonstrate SNS feasibility in these patients.

**Methods:**

This retrospective study includes patients who received a SNS between 1993 and 2020 for increased stool frequency or fecal incontinence after proctocolectomy with IPAA for ulcerative colitis. Proctocolectomy was performed in a two- or three-step approach with ileostomy closure as the last step. Demographic, follow-up data and functional results were obtained from the hospital database.

**Results:**

SNS was performed in 23 patients. Median follow-up time after SNS was 6.5 years (min. 4.2–max. 8.8). Two patients were lost to follow-up. The median time from ileostomy closure to SNS implantation was 6 years (min. 0.5–max. 14.5). Continence after SNS improved in 16 patients (69%) with a median St. Marks score for anal incontinence of 19 (min. 4–max. 22) before SNS compared to 4 (0–10) after SNS placement (p = 0.012). In seven patients, SNS therapy was not successful.

**Conclusion:**

SNS implantation improves symptoms in over two-thirds of patients suffering from high stool frequency or fecal incontinence after proctocolectomy with IPAA. Awareness of the beneficial effects of SNS should be increased in physicians involved in the management of these patients.

## Introduction

J-pouch formation with ileal pouch-anal anastomosis (IPAA) is the restorative procedure of choice after proctocolectomy [1]. Functional results after IPAA are usually very good [2–4]. However, fecal incontinence may be a consequence and negatively impact quality of life (QoL) and overall outcome. Fecal incontinence is defined as the inability to control feces or gas. The range of fecal incontinence described after IPAA varies widely and seems to progress over time [3–5]. Daytime stool incontinence is described in 1 to 25% of patients after J-pouch formation with IPAA [2–7]. Incontinence at night seems to be more common at 8 to 49% [3–8]. Increased stool frequency is another reported factor that negatively impacts quality of life [9].

The pathogenesis of fecal incontinence after J-pouch reconstruction with IPAA is not fully understood. On the one hand, proctocolectomy with IPAA includes thorough intersphincteric excision and, on the other hand, maximum preservation of the sphincter function. Even so, the procedure can affect the upper parts of the internal sphincter and therefore may cause dysfunction, mainly at night [8]. Another possible mechanism could be a delayed function of the puborectalis muscle due to an altered postoperative latency of the sacral nerve [10].

Sacral nerve stimulation (SNS) is an established therapy for fecal incontinence in general or in patients with sphincter defects [11, 12] with low perioperative morbidity and good patients’ experience [12, 13]. Recent experience has shown good feasibility and clinical benefit, albeit based primarily on retrospective data [14, 15]. In addition, SNS seems to be also effective in the treatment of functional deficits after IPAA [2, 16]. These observations are encouraging but are based on studies with a small number of patients [2]. There is a case report (n = 1) [17], a retrospective study (n = 7) [18], and a cohort study (n = 4) [16] working on SNS for IPAA. We, therefore, analyzed our experience from over 20 years of IPAA surgery and SNS. With this study, we aimed to evaluate the effectiveness and usefulness of SNS therapy for the treatment of fecal incontinence according to IPAA.

## Methods

### Patients

This is a retrospective single-center, single-cohort outcome study evaluating the effect of SNS in patients with high stool frequency or fecal incontinence after proctocolectomy with J-pouch reconstruction and IPAA. Inclusion period was from January 1993 until December 2020. The analysis included gender, age, time to SNS, digital rectal examination scoring system (DRESS) before and after SNS implantation, St. Marks incontinence score (validated score for fecal incontinence) before and after SNS implantation, fecal incontinence at night, and stool thickening drugs before and after SNS implantation, as well as biofeedback and pelvic floor training as further continence supporting tools before and after SNS implantation.

## Interventions

The indication for SNS implantation in patients with refractory fecal incontinence treatment after IPAA was based on individual decisions and is currently not an established therapeutic method, including in this work. All selected patients initially received a PNE (percutaneous nerve examination) as part of a test phase. Since all patients showed a positive signal to PNE, a temporary stimulating electrode was placed under general anesthesia. Based on clinical standards, prophylactic antibiotics were given prior to the procedure. During electrode insertion, radiological controls of correct placement close to sacral foramen two (S2) or three (S3) and electrophysiological monitoring of contractions of the pelvic floor, the external anal sphincter, and the toe were performed. Optimal electrode position was defined as the best motor response of the pelvic floor/external sphincter muscle with low stimulation amplitude. The PNE line then was attached to the skin. PNE was started 4 to 12 h after intervention. After hospital discharge and with a successful response and symptom improvement of at least 50%, the final SNS was implanted. If improvement was less than 50%, PNE were removed. For final implantation, a tined quadripolar lead was inserted into the same sacral foramen and evaluated under radiological and electrophysiological monitoring as with the PNE. The electrode was then leaded subcutaneously and connected with an implantable pulse generator (IPG, Medtronic, Interstim 3625) according to the manufacturer’s recommendations. The IPG was implanted and fixed subcutaneously in the gluteal area, and the wound was closed in two-layer suture. Stimulation was started 12 h after intervention with a program configuration according to patient’s sensory response. Wound scaring, local pain, and therapy success were controlled 2 and 6 weeks after the procedure.

## Statistics

Since most variables showed skewed distributions, non-parametric tests were used for statistical comparison. Continuous variables are displayed as median (minimum–maximum), and categorical variables are displayed as count (percentage). ASA is displayed as mean (minimum–maximum) to allow for a finer graduation. The Mann–Whitney U test was used to compare two independent groups. For dependent variables, the Wilcoxon test was used. The chi-square test was used for group comparisons of categorical variables. The level of significance was 0.05 (two-sided) for each statistical testing. The statistical analysis was performed with SPSS Statistics Software 25.0 (IBM, Armonk, NY, USA).

## Ethics

The Medical Ethical Committee of Charité — Universitätsmedizin Berlin (EA1/289/20) approved the study protocol.

## Results

### Demographics

A total of 23 patients underwent SNS implantation for fecal incontinence or high stool frequency (Table 1). Sixty-nine percent of these patients (n = 16) were successfully treated with SNS. In 7 patients (31%), the test electrode had to be removed as the improvement was below 50%. Median follow-up time was 6.5 years. Two patients were lost to follow-up.

**Table 1:** Demographics

	N = 23
Age, years	53 (27–69)
Sex, male	15 (65)
BMI, kg/m^2^	23.5 (18.8–38.9)
Multi-stage procedure	
Two-stage	16 (74)
Three-stage	7 (26)
IPAA	
Hand-sewn	21 (93)
Stapled	2 (7)
Time to SNS, years	6 (0.5–14.5)
Successful SNS	16 (69)
Follow-up, years (95% CI)	6.5 (4.2–8.8)

*BMI *body mass index, *IPAA* ileal pouch anal anastomosis, *SNS* sacral nerve stimulation, Time to SNS = time between IPAA and SNS. Median (min–max) for continuous variables, count (percent) for categorical variables

### Possible factors influencing the success of SNS

Of our patients, 74% had a two-stage procedure, and 26% had a three-stage procedure (Table 2). The choice of procedure had no significant influence on the success rate (p = 0.618). Patients with frustrated SNS implantation had a significantly higher BMI in contrast to successful SNS (30.2 kg/m^2^ vs. 22.8 kg/m^2^; p = 0.008). In addition, we examined the type of IPAA (hand-sewn vs. stapled), whereby the majority of IPAAs (91%) were hand-sewn, so that no statistical differences could be found. The median time from IPAA to SNS was 6.7 years for successful SNS and 2.2 years for non-successful SNS, but with no significant difference (p = 624).

**Table 2:** Possible factors influencing the success of SNS

	Success N = 16	No success N = 7	Missing	P-value
Age, years	53 (27–69)	53 (30–63)	-	0.769
Sex, male	11 (69)	4 (57)	-	0.467
BMI, kg/m^2^	22.8 (18.8–27.4)	30.2 (24.9–38, 39)	9	0.008
Multi-stage procedure			-	0.681
Two-stage	12 (75)	5 (71)		
Three-stage	4 (25)	2 (29)		
IPAA, hand-sewn	15 (94)	6 (86)	-	0.526
IPAA, stapled	1 (6)	1 (14)		
Time to SNS, years	6.7 (1.2–14.4)	2.2 (0.5–14.5)	-	0.624

*BMI* body mass index, *IPAA* ileal pouch anal anastomosis, *SNS* sacral nerve stimulation, Time to SNS = time between IPAA and SNS. Median (min–max) for continuous variables, count (percent) for categorical variables

### Successful SNS implantation

Fecal incontinence at night significantly improved after SNS implantation (Table 3). However, SNS did not affect stool frequency in general. Table 4 shows a significant improvement in the St. Marks incontinence score from 19 to 2 in patients with successful SNS implantation (p = 0.012). The amount of continence supporting tools (biofeedback, pelvic floor training) and antidiarrheal agents did not change after SNS implantation.

Table 5 shows all 16 successful SNS implantations. Median follow-up in this patient group was 5.1 years (1.7–8.4). One patient was lost to follow-up. Median time from IPAA to SNS was 6.7 years (1.2–14.4). Despite missing data due to lack of documentation, St. Marks incontinence score and nocturnal incontinence at night were better for most patients.

**Table 3:** Results after 16 successful SNS implantations

	Before SNS N = 16	Missing	After SNS N = 16	Missing	P value
Stool frequency	9 (8–18)	11 (68)	6 (3–6)	13 (81)	0.102
Nocturnal fecal incontinence	6 (38)	10 (62)	5 (31)	11 (68)	0.025
St. Marks incontinence score	19 (4–24)	6 (37)	4 (0–18)	3 (19)	0.012
DRESS Score	2 (0–4)	8 (50)	3 (2–4)	2 (13)	0.414
Continence supporting tools					
Biofeedback	5 (31)	5 (31)	0	1 (6)	0.739
Pelvic floor training	3 (19)	5 (31)	0	1 (6)	0.257
Antidiarrheal agents					
Loperamide	9 (56)	5 (31)	10 (63)	1 (6)	0.083
Tincture of opium	2 (13)	5 (31)	1 (6)	1 (6)	0.206
Apple fruit extract	3 (19)	5 (31)	1 (6)	1 (6)	0.366

St. Marks incontinence score (min. = 0, max. = 24). DRESS Score (= digital rectal examination scoring system; min. = 0, max. = 5). Median (min–max) for continuous variables, count (percent) for categorical variables

**Table 4:** Differences in incontinence before and after SNS (St. Marks incontinence score)

	Before SNS	After SNS	P-value
Successful SNS	19 (4–22)	2 (0–10)	0.012
Non-successful SNS	20 (12–24)	20 (0–24)	0.317

St. Marks incontinence score (min. = 0, max. = 24) *SNS* sacral nerve stimulation, Time to SNS = time between IPAA and SNS

**Table 5:** Demographics and results of all 16 successful SNS implantation

	Age/sex	Time to SNS, years	Stool frequency before/after SNS	Nightly incontinence before/after SNS	DRESS before/after SNS	St. Marks score before/after SNS	Follow-up, years
Patient 1	49/male	7.6	-/-	-/-	3/2	-/-	0.1
Patient 2	65/female	6	-/-	-/-	-/3	-/10	10.7
Patient 3	50/male	5	-/-	-/-	-/3	-/18	7.8
Patient 4	54/male	1.8	-/-	-/-	-/3	-/18	6.8
Patient 5	52/male	8.9	-/-	-/-	-/3	-/0	8.5
Patient 6	51/male	13.1	-/-	-/-	-/3	-/4	6.5
Patient 7	44/female	5.6	-/-	-/-	-/3	20/4	3.3
Patient 8	59/male	2	8/-	1/0	3/3	24/-	2.3
Patient 9	42/male	12.8	-/-	-/-	4/4	14/2	5.1
Patient 10	62/male	14.4	-/-	-/-	3/2	4/2	5.1
Patient 11	27/female	1.3	-/-	-/-	2/3	12/2	2.9
Patient 12	54/female	7.9	18/6	1/0	-/-	20/0	3.4
Patient 13	59/male	7.5	11/6	1/0	2/2	20/4	0.3
Patient 14	69/male	13.8	8/3	1/0	2/2	18/0	4.0
Patient 15	44/male	2.3	9/ -	1/0	-/-	22/10	8.7
Patient 16	54/female	1.2	-/-	1/-	0/2	10/-	0

Time to SNS *= *time from IPAA to SNS. DRESS Score (= digital rectal examination scoring system; min. = 0, max. = 5) *IPAA* ileal pouch anal anastomosis, *SNS* sacral nerve stimulation

### Failed SNS implantation

Table 6 shows demographics and results of all seven failed SNS implantations. After a control period of at least 3 months, test electrodes were removed when the procedure was not successful. The pouch was removed in three patients due to severe pouchitis, persistent incontinence, or severe anal pain. Two patients received an ostomy again because of persistent incontinence. Two patients were lost to follow-up.

**Table 6:** Demographics and results of all 7 failed SNS implantation

	Age/sex	Time to SNS, years	Stool frequency before/after SNS	Nightly incontinence before/after SNS	DRESS before /after SNS	St. Marks score before/after SNS	Follow-up, years	Clinical course
Patient 1	30/female	1.4	9/9	1/1	2/2	20/20	0.5	Anal pain, stenotic IPAA, pouch explantation
Patient 2	30/male	0.5	-	-/-	2/2	25/25	0.01	Severe pouchitis resulted in Pouch explantation
Patient 3	63/male	1.1	12/10	-/-	-/-	12/0	7.5	Persisting incontinence, new stoma
Patient 4	57/male	9.4	8/-	1/-	-/-	8/25	0	Patient lost to follow-up
Patient 5	53/male	13.3	14/14	1/1	1/1	20/20	10.9	Persisting incontinence, Pouch explantation
Patient 6	50/female	14.5	20/20	1/1	1/1	24 /24	0.05	Patient lost to follow-up
Patient 7	46/female	2.2	8/8	1/1	0/0	16/16	0.2	Persisting incontinence, new stoma

Time to SNS = time from IPAA to SNS. DRESS Score (= digital rectal examination scoring system; min. = 0, max. = 5) *IPAA* ileal pouch anal anastomosis, *SNS* sacral nerve stimulation

## Discussion

The aim of our study was to evaluate the feasibility and the benefit of sacral nerve stimulation (SNS) in the treatment of fecal incontinence in patients with J-pouch reconstruction and ileal pouch-anal anastomosis (IPAA).

Our study shows the feasibility of SNS in IPAA patients by improving fecal incontinence (as measured by the St. Marks incontinence score) in more than two-thirds of patients with fecal incontinence or high stool frequency. The St. Marks incontinence or Vaizey score was used as a rating system for the assessment of the severity of fecal incontinence [19]. Our results showed a significant improvement after SNS implantation from 19 to 4 in the St. Marks incontinence score. There is some evidence that shows a positive correlation between St. Marks incontinence score and quality of life [20, 21]. With decreasing severity of the St. Marks incontinence score, the quality of life measured with the Manchester Health Questionnaire (MHQ) was improved [21]. Due to this positive correlation, indirect conclusions can also be drawn about a better quality of life observed in our patient cohort after successful SNS implantation. In our study, we were able to demonstrate an improvement in nighttime incontinence after SNS. Although some data were missing for the evaluation, this may indicate a possible improvement in quality of life after SNS for these patients.

In the analysis of possible factors influencing the success of SNS, a significantly higher BMI of 30.2 kg/m^2^ (24.9–38.9) was found for non-successful SNS. However, the statistical significance of BMI on success after SNS must be taken with caution in view of the missing data. The data lack is mainly due to the conversion of the patient recording system from analog to digital over the years. However, it is consistent with the literature that there are more perioperative problems with IPAA in obese patients [22, 23].

Increased stool frequency is another reported factor that negatively impacts quality of life [9]. However, overall stool frequency was not affected by SNS implantation in this analysis. This is reflected in previous work describing low daytime fecal incontinence of 1 to 25% in contrast to nighttime incontinence of 8 to 49% [2–8].

In seven patients, the therapy had to be discontinued after the test phase and the electrodes removed because the SNS implantation was not successful. In some of these patients, a new stoma or even a pouch explantation had to be performed. Postoperative pouchitis or fistulas could be a cause of reduced pouch function. As described in the literature, chronic pouchitis, fistulas, leakage, or conversion to a diagnosis of Crohn’s disease pose a clear risk for failure of the J-pouch and IPAA [24, 25]. However, some patients had persistent incontinence for no apparent reason. Further investigations seem necessary to clarify the causes of SNS failure.

A limitation of this study was the partially incomplete data collection. On the one hand, this is due to the very long study period of 20 years with a certain loss of data due to the conversion of the documentation from analog to digital. On the other hand, the follow-up data are missing because they were either collected in the patients’ hometowns or not at all. A retrospective survey might have yielded more results. Our results on stool frequency and nocturnal incontinence must therefore be interpreted cautiously, as the number of missing values was quite high.

However, the strength of our work lies in the relatively large number of patients and the long follow-up period with an average of 6 years. As there are only single case reports and small studies in the literature so far, it is now possible to make more comprehensive statements about the success of SNS use for fecal incontinence after IPAA.

## Conclusions

SNS implantation for the treatment of fecal incontinence and high stool frequency in patients with J-pouch and ulcerative colitis shows good results. SNS appears to be a good therapy option for these patients and should be considered at an early stage and may offer an alternative to J-pouch removement or permanent stoma application.

## Data Availability

The data that support the findings of this study are available from the corresponding author upon reasonable request.
